# Trigeminal somatosensory evoked potentials for intraoperative monitoring and prognostic prediction in microvascular decompression for trigeminal neuralgia: a prospective cohort study

**DOI:** 10.3389/fneur.2025.1693948

**Published:** 2026-01-29

**Authors:** Yan Zhang, Haiguang Liu, Yuanyuan Zhang, Yuanbo Hu, Yanbin Wang, Haipeng Xie, Xichao Wen, Wensong Wu, Zetong Bai, Kebin Zheng

**Affiliations:** 1 Baoji Central Hospital, Shaanxi, China; 2 Affiliated Hospital of Hebei University, Baoding, China; 3 Clinical Medicine College, Hebei University, Baoding, China; 4 College of Basic Medical Sciences, Hebei University, Baoding, China

**Keywords:** microvascular decompression, primary trigeminal neuralgia, prognosis, trigeminal somatosensory evoked potentials, waveform change rate

## Abstract

**Objective:**

This study aims to explore the monitoring techniques of trigeminal somatosensory evoked potentials (TSEP) and investigate their clinical significance in microvascular decompression (MVD) for primary trigeminal neuralgia (PTN). By analyzing the relationship between changes in TSEP waveforms during surgery and postoperative outcomes of MVD, a prognostic prediction model based on electrophysiological and clinical features will be constructed to provide a basis for individualized assessment of surgical risks and efficacy.

**Methods:**

This study included 74 patients diagnosed with PTN and used statistical methods to analyze and optimize the waveform characteristics of TSEP. The amplitude change rate of TSEP before and after MVD surgery was calculated, and combined with postoperative pain scores, Spearman correlation analysis and ROC curves were used to determine the optimal cut-off value for predicting prognosis. Finally, univariate and multivariate Cox regression analyses were conducted to identify independent risk factors affecting poor prognosis of MVD surgery in PTN patients, a nomogram model was established, and the model’s performance was validated through Kaplan–Meier survival analysis and ROC curves.

**Results:**

(1) In this study, there was no statistically significant difference in the amplitudes of V1w1 and V1w2 between the healthy and affected sides in TSEP (*p* > 0.05), while the amplitudes of TNW1, TNW2, TNW3, V2w1, V2w2, V2w3, V3w1, V3w2, and V3w3 showed significant statistical differences (*p* < 0.05); the latencies of all TSEP branches showed no significant statistical differences before and after MVD surgery (*p* > 0.05). (2) The TSEP amplitude change rates were calculated, and the amplitude change rate of TNW2 was strongly negatively correlated with postoperative pain, with TNW2 showing the strongest correlation [*r* = −0.563, *p* < 0.05], followed by TNW3. (3) ROC curve analysis of the relationship between TNW2, TNW3, and surgical prognosis indicated that both could predict surgical outcomes (*p* < 0.05): TNW2 [AUC = 0.792, Cut-off = 1.595, i.e., 59.5%]; TNW3 [AUC = 0.760, Cut-off = 1.535, i.e., 53.5%]. (4) Cox proportional hazards regression analysis identified independent risk factors affecting surgical prognosis. Multivariate analysis showed that TNW2 amplitude change rate [HR = 0.27, 95% CI: 0.11–0.67, *p* = 0.005], hypertension [HR = 0.54, 95% CI: 0.30–0.97, *p* = 0.039], and PTN disease course [HR = 0.47, 95% CI: 0.24–0.90, *p* = 0.023] were independent prognostic factors. The nomogram model had AUC values of 0.80, 0.83, and 0.93 at 14, 30, and 90 days, respectively, showing good discrimination. Kaplan–Meier analysis further confirmed the significant association of TNW2 amplitude change rate, hypertension, and PTN disease course with prognosis (Log-rank *p* < 0.001).

**Conclusion:**

The TSEP technique used in this study is simple to operate (only requiring puncture around the muscle groups near the puncture point), provides stable waveform results, and is convenient for intraoperative interpretation (only needing to observe changes in waveform amplitude). In addition, intraoperative monitoring of TNW2 amplitude change rate (≥60% indicates good prognosis) can provide real-time guidance during decompression and predict efficacy. The combination of hypertension, PTN duration ≥2.5 years, and TNW2 amplitude change rate <60% are independent risk factors for poor prognosis after MVD; patients with PTN who have hypertension, longer PTN duration, or TNW2 amplitude change rate <60% during microvascular decompression experience slower postoperative pain relief. The nomogram model based on TSEP waveforms, hypertension, and PTN duration has high accuracy and can individually assess recurrence risk, providing a tool for clinically identifying high-risk patients.

## Introduction

The trigeminal nerve (V) is the fifth cranial nerve and is responsible for detecting sensory stimuli from the craniofacial region. It is divided into three branches: ophthalmic (V1), maxillary (V2), and mandibular (V3). Trigeminal neuralgia (TN) is known as the “most painful condition in the world” and severely affects people’s quality of daily life (1–3). There are different theories regarding the etiology of primary trigeminal neuralgia, with nerve and blood vessel compression (NVC) being the most widely accepted theory. In 1945, Walter Dandy and others first proposed that TN is caused by compression or abnormal distortion of the superior cerebellar artery at the root entry zone (REZ) of the trigeminal nerve, and microvascular decompression surgery was developed based on this theory. With advantages such as being minimally invasive, safe, and effective, it quickly became the most effective surgical method for treating primary TN (4–6).

Trigeminal sensory evoked potentials (TSEP) can reflect the functional level and excitability of the trigeminal nerve itself, trigeminal nuclei, and central conduction pathways. By stimulating any location in the distribution area of the peripheral branches of the trigeminal nerve, a series of evoked potentials can be recorded in the corresponding cortical sensory areas (7, 8). Larsson et al. first reported TSEP in 1970. In the early stages of research, the recorded TSEP lasted 10–50 ms, the waveform generation was poor and unstable, and it was easily affected by electromyography and intraoperative medications, which seriously affected its clinical application and research value. Numerous subsequent studies on TSEP have gradually improved monitoring methods and techniques, making it possible to record stable subcortical evoked potentials within 10 ms (9, 10). Leandri et al. reported in 1985 that there are three main waveforms within 10 ms, primarily W1, W2, and W3. W1 is a high-amplitude triphasic positive wave, while W2 and W3 are low-amplitude monophasic negative waves. In 1987, further studies based on TSEP were conducted using the NCR lead, detecting four small waves—P4, N5, P6, and N7—following the W3 wave (7, 8). This has deepened people’s understanding of TSEP.

With the ongoing in-depth research on TSEP, the currently widely used TSEP waveforms are clear, stable, reproducible, and show minimal differences in latency. Even when using preoperative muscle relaxants, general anesthetics, or when the patient is drowsy, has altered consciousness, or other EEG activities occur, the presynaptic waveforms (W1, W2, W3) can still be clearly distinguished (11, 12). Although there are significant differences in TSEP monitoring methods and results reported in different studies, leading to a lack of standardized monitoring protocols and interpretation criteria, there is general consensus regarding the neural origins of each TSEP wave. The W1 wave is considered to arise from the trigeminal ganglion of the trigeminal nerve, the W2 wave from the REZ, the W3 wave from the trigeminal sensory principal nucleus, N5 from the trigemino-thalamic tract, and P6 from the thalamus. The key research focus includes the W2 wave, which reflects the electrical conduction in the trigeminal nerve REZ, and the W3 wave, which reflects electrical changes in the trigeminal sensory principal nucleus. The time difference between the peaks of W1 and W2 reflects the functional conduction of the trigeminal peripheral branches; the time difference between the peaks of W2 and W3 reflects the conduction function from the REZ area to the trigeminal sensory main nucleus (7, 13, 14). In patients with primary trigeminal neuralgia (PTN), common TSEP abnormal waveforms usually appear in two forms: disappearance of W2 and W3 waves, or poor waveform differentiation. Of course, waveform characteristics vary among different types of PTN patients. Poorly differentiated waveforms are mainly manifested as W2 and W3 waves being indistinguishable, replaced instead by a large negative waveform, with longer latency and duration. Moreover, when the trigeminal REZ area receives adequate decompression, trigeminal conductive function returns to normal or mostly normal, manifested as the recovery of W2 and W3 wave latency, delayed recovery, or temporary fusion of W2 and W3 waves (10).

The TSEP operation method used in this study is simplified, no longer requiring the stimulation electrodes to be precisely inserted into the supraorbital or infraorbital foramina; it is sufficient to insert them into the surrounding muscle groups to obtain stable and clear waveforms. Moreover, during monitoring, it is not necessary to comprehensively analyze the latencies and amplitudes of each TSEP wave or calculate the waveform area to assess decompression; it is enough to focus on the trend of changes in wave amplitudes to determine whether decompression is adequate and predict the prognosis. Additionally, postoperative assessment of decompression adequacy can be guided solely by the amplitude changes of the total branch W2 and W3 waves, or of the total branch W2 wave, or the total branch W3 wave. This can then be combined with whether the patient has hypertension, the duration of PTN, and other factors to predict the patient’s postoperative recovery.

## Methods

### Study population

This study screened patients diagnosed with PTN who visited the Department of Neurosurgery at the Affiliated Hospital of Hebei University from January 2024 to October 2024. Inclusion criteria included patients diagnosed with PTN confirmed by a senior neurosurgery specialist (Z.K.B) before the start of the study and who underwent MVD surgery with bilateral TSEP monitoring. Diagnostic criteria followed the International Classification of Headache Disorders (ICHD-3) for confirming PTN, with clinical symptoms and auxiliary examination results meeting the diagnostic standards outlined in the “Expert Consensus on the Diagnosis and Treatment of Trigeminal Neuralgia in China.” Exclusion criteria included a history of cranial surgery, secondary trigeminal neuralgia, recurrent trigeminal neuralgia, other types of pain disorders, and psychiatric diseases. Elimination criteria included patients with poor monitoring waveform differentiation, intraoperative anesthetic doses exceeding 15%, and intraoperative use of muscle relaxants. Dropout criteria applied to patients lost to follow-up for various reasons.

All enrolled patients were assessed for pain using the Visual Analogue Scale (VAS) before surgery, and the onset time of the disease along with other general information was recorded. All patients were followed up once a month, and the VAS pain scale was used again during each follow-up to assess pain. The total follow-up period was 3 months.

At the time of follow-up enrollment, we collected each patient’s predictive factors based on previous studies or clinical characteristics, which may be related to the postoperative prognosis of MVD in PTN patients. Demographic data, including age and gender, were recorded. Past medical history was obtained from medical records or provided by the patient, including whether they had hypertension, duration of hypertension, diabetes, and cerebral infarction. Clinical characteristics were determined by the onset features of PTN, including the location of pain (ophthalmic branch, maxillary branch, mandibular branch, or multiple regions), duration of PTN, and left or right side involvement. The severity of pain was assessed using the VAS pain scale, which was conducted both before surgery and during postoperative follow-up.

### Monitoring instruments and materials

Electrophysiology testing device: CADWELL electrophysiology monitoring device from the USA (model: Cascade).

### Stimulation and recording electrodes

(1) Stimulation Electrode: Medical disposable needle electrode (stranded wire) (Model: NE-T-2500/13/0.4, Xi’an Fude Medical Electronics Co., Ltd.) Specifications: needle length 13 mm/needle diameter 0.4 mm/wire length 2.5 m.
(2) Recording Electrode: Medical disposable needle electrode (single wire) (Model: NE-S-2500/13/0.4, Xi’an Fude Medical Electronics Co., Ltd.) Specifications: Needle length 13 mm/Needle diameter 0.4 mm/Wire length 2.5 m.

### Puncture method

*Ophthalmic branch (V1)*: The puncture point for the ophthalmic branch is located in the medial one-third of the eyebrow surrounding muscle group (i.e., the muscles around the supraorbital notch). When puncturing, the stimulating electrode is inserted obliquely into the muscle tissue to stimulate the ophthalmic branch of the trigeminal nerve.

*Maxillary branch (V2)*: The puncture point for the maxillary branch is located at the junction of the outer lateral third of the orbicularis oris muscle and the outer lower part of the ala of the nose; during puncture, the stimulating electrode is inserted at an angle to stimulate the maxillary branch of the trigeminal nerve.

*Mandibular branch (V3)*: The puncture point for the mandibular branch is located in the muscle group around the mandibular angle. During puncture, the stimulation electrode is inserted at an angle to stimulate the mandibular branch of the trigeminal nerve.

*TN*: The puncture point for the TN branch is located at the zygomatic foramen. During the puncture, it can be inserted vertically to stimulate the zygomatic nerve.

*C3’*: The recording electrode C3’ is located 1 cm anterior to C3.

**Figure 1:**
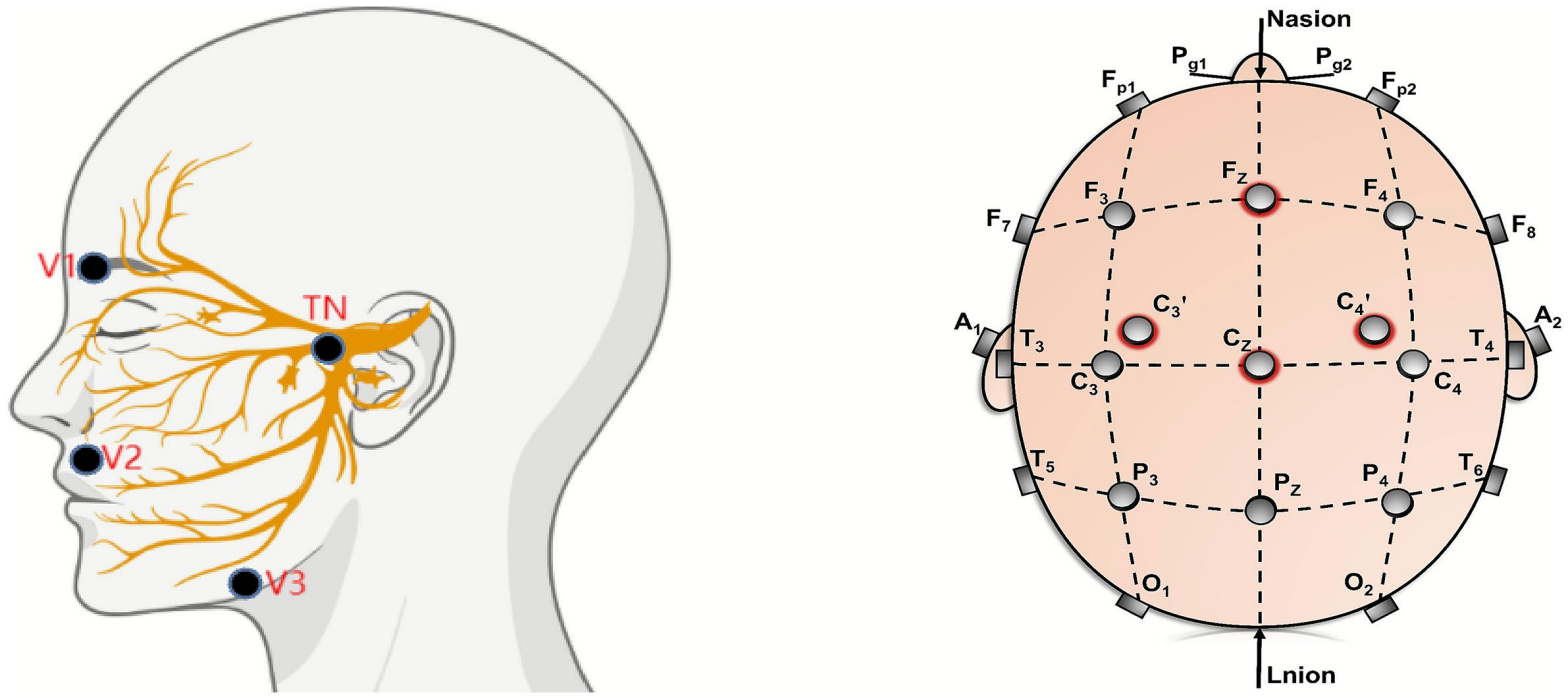
TSEP puncture point diagram.

*C4’*: The recording electrode C4’ is located 1 cm in front of C4; (Figure 1).

### Stimulation requirements and parameter settings

(1) Stimulation requirements: Maintain room temperature at 18–25 °C. Use Leandri’s needle electrode stimulation method, with the stimulating electrode (twisted wire) inserted into the corresponding stimulation sites of the ophthalmic, maxillary, mandibular, and trigeminal nerve branches to stimulate the trigeminal nerve V1, V2, V3, and the main trunk; the distance between stimulating electrodes is 1 mm, electrodes do not cross each other, and the insertion depth is 10 mm. The recording electrode (single wire) is inserted at the midpoint of the top of the head (CZ), the ground electrode (single wire) is inserted at the midpoint of the forehead (FPz), and the reference electrode (single wire) is inserted at the spinous process of the 7th cervical vertebra (CV7).
(2) Stimulation parameters: The stimulation analysis time is 10 ms, pulse width 0.05 ms, pulse duration 200 μs, stimulation frequency 2.1 Hz, filter 30–500 Hz, waveform signal superimposed 500 times,amplifier input 1,600 μV, skin-to-skin impedance <5 kΩ. Connection of Electrodes to Equipment.

### Electrode and device connection

(1) Connection of electrodes to the stimulator: The stimulation electrodes at the left ophthalmic branch, maxillary branch, and mandibular branch connect to points 1, 3, and 5, respectively; the stimulation electrodes at the right ophthalmic branch, maxillary branch, and mandibular branch connect to points 2, 4, and 6, respectively.
(2) Connection of electrodes to the recording board: The recording electrode (Cz) connects to point 1, the reference electrode (Cv7) connects to point 4, and the ground electrode (Fpz) has a dedicated interface.
(3) After all electrodes are connected, measure the skin impedance, which should be <5 kΩ.

### Stimulation methods and recording content

Stimulation method: All stimulations were performed under general anesthesia. After anesthesia was administered, puncture was performed; all patients discontinued muscle relaxants once the surgery officially began. TSEP detection was carried out before the start of surgery, with the stimulation intensity gradually increased from 0 mA, increasing by 0.5 mA each time, and recordings were made simultaneously on both sides until a stable TSEP could be recorded.

Recording details: Before the surgery began, the TSEP waveforms of the ophthalmic, maxillary, mandibular, and total branches on both sides were recorded. During monitoring, facial muscle contractions in response to increasing stimulation intensity were continuously observed to understand whether muscle contractions interfered with the waveforms.

### Measurement method

Amplitude measurement method: Methods for measuring the amplitudes of waveforms W1, W2, and W3. Take the starting point of W1, W2, and W3 as the baseline respectively, and measure the distance from the baseline to the waveform peak. The measurement tool is the latency and amplitude measurement software included in the electrophysiological monitoring system.


$$\begin{array}{l} \text{Waveform change rate}=(\begin{array}{l} \text{postoperative amplitude absolute value}\\ -\text{preoperative amplitude absolute value} \end{array})\\ /\text{preoperative amplitude absolute value}. \end{array}$$

### Statistical analysis

All statistical analyses were performed using the SPSS (Statistical Package for the Social Sciences) software package (version 26.0). The Shapiro–Wilk test was used to assess the normality of each quantitative variable. Quantitative data are presented as mean ± standard deviation, while categorical variables are presented as percentages. Quantitative data were analyzed using one-way analysis of variance (ANOVA) or the Mann–Whitney U test. The pre- and post-operative waveform change rates of TSEP were calculated, and Spearman correlation analysis was used to examine the relationship between TSEP waveform change rates and postoperative pain scores. ROC curve analysis was again used to determine the optimal cutoff value and identify the best waveform change rate.

Cox regression analysis was used to examine the risk factors affecting the prognosis of MVD surgery in PTN patients. First, univariate Cox regression analysis was performed on all baseline variables, followed by stepwise variable selection in multivariate Cox regression. The significance levels for entry and retention were set at 0.1 to avoid excluding potentially important variables. For other statistical analyses, a two-sided *p*-value < 0.05 was considered statistically significant. After screening relevant factors based on multivariate logistic regression, a predictive model was constructed using a nomogram. The analysis was conducted using the ‘rms’ package in R Studio (R Foundation for Statistical Computing, Vienna, Austria). The predictive ability of the model was evaluated through calibration curves, and the stability of the predictive model was validated using the ROC curve. Finally, Kaplan–Meier survival analysis was performed to further verify the correlation of three factors—TNW2 amplitude change rate, hypertension, and PTN disease course—with MVD surgical prognosis.

### Surgical technique

All patients diagnosed with PTN underwent microvascular decompression (MVD) (14), performed by the same surgeon. The surgical procedure involved the following steps: the patient was placed in a supine position on the unaffected side, and general anesthesia was administered. A transverse incision approximately 3 to 4 cm in length was made behind the ear and within the hairline on the affected side, with the sigmoid sinus serving as the lateral limit and the transverse sinus as the superior limit of the bone window. The dura mater was incised in a flap-like manner, and a microscope was inserted. The upper part of the posterior cranial fossa and the trigeminal nerve root were fully exposed, and cerebrospinal fluid was released. The cerebellar hemisphere was gradually retracted using a brain retractor, and the petrosal vein was dissected. The arachnoid membranes attached to the nerve root and the brain stem were gradually cut away. The nerve root and the responsible artery were freed using microdissection forceps and separated with Teflon sheets. The dura mater was sutured, and the cranial closure was performed.

### Postoperative management

**Figure 2:**
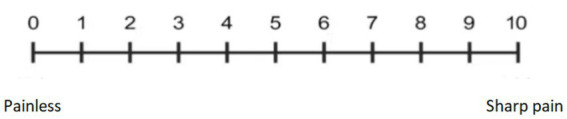
Visual analogue pain scale (VAS).

After surgery, all patients need to be transferred to our general inpatient ward for observation and receive symptomatic medication treatment. Routinely, a cranial CT scan is arranged within 24 h post-surgery to confirm the presence of potential complications such as intracranial hemorrhage. Additionally, during their hospital stay, patients are assessed daily using the VAS pain scale (Figure 2) to monitor changes in postoperative pain. Follow-up visits will be conducted at 1, 2, and 3 months after discharge to assess the patients’ recovery progress.

## Results

### Demographic and clinical characteristics

In this study, a total of 74 patients with PTN were included who met the study eligibility criteria. Among the study cohort, there were 23 male patients (32.0%) and 51 female patients (68%); the average baseline age was 58.3 ± 11.5 years; 43 patients (58.1%) had a history of hypertension, with an average duration of 3 ± 3.6 years; 10 patients (13.5%) had diabetes; 9 patients (12.2%) had a history of cerebral infarction; 21 patients (28.4%) had left facial pain, and 53 patients (71.6%) had right facial pain; the average preoperative pain score was 7.6 ± 1.5; the average duration of illness (defined as the time from onset to registration) was 4.7 ± 2.8 years, and 64 patients (86.5%) had good postoperative follow-up outcomes (Table 1).

**Table 1:** Baseline characteristics of enrolled patients.

General (*N* = 74)	
Female	51(68%)
Age (years)	58.3 ± 11.5
Height	165.5 ± 7.6
Weight	58.9 ± 7.4
Hypertension	43(58.1%)
Hypertension history (years)	3 ± 3.6
Diabetes	10(13.5%)
Stroke	9(12.2%)
Right-sided pain	53(71.6%)
Preop VAS score	7.6 ± 1.5
PTN duration (years)	4.7 ± 2.8
Favorable outcome	64(86.5%)

### TSEP monitoring results

**Figure 3:**
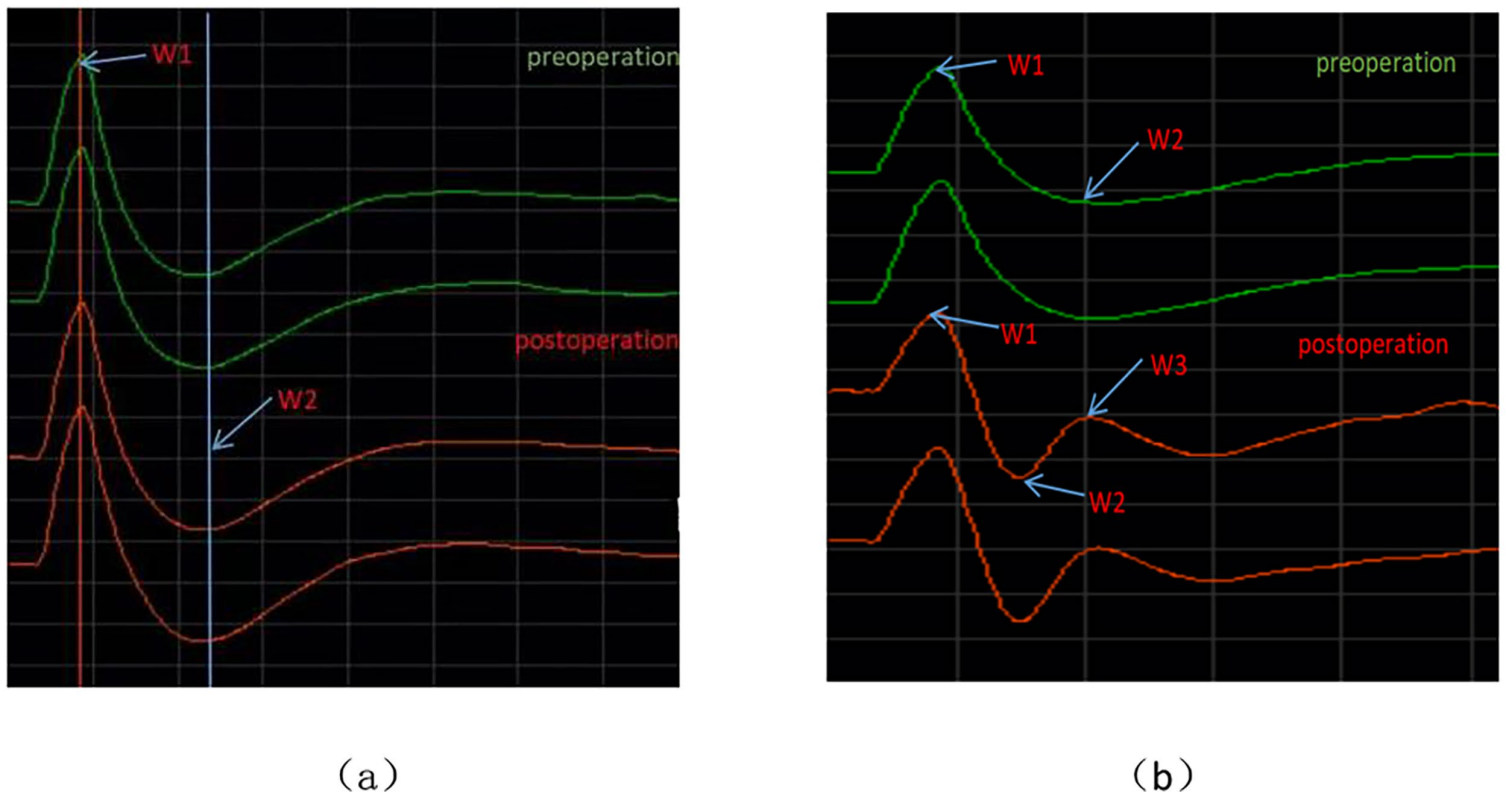
Waveform charts before and after TSEP. **(a)** Represents the schematic diagram of latency measurement; **(b)** represents the schematic diagram of amplitude measurement.

During TSEP monitoring, the stimulation intensity for the main branch, V1 branch, V2 branch, and V3 branch all started from 0 mA and gradually increased. When below 1 mA, the waveforms could not be differentiated. At a stimulation intensity of 1–15 mA, although the TSEP was well differentiated, waveform changes at different times were minimal. When the stimulation intensity gradually increased to above 20 mA, noticeable waveform changes could be observed in W1, W2, and W3 at different times. W1 is the first differentiated positive wave, with occasional W1 wave variations, presenting as biphasic or triphasic waves, but these do not interfere with the identification of W2 and W3 waveforms. W2 is the first differentiated negative wave, with occasional W2 wave variations, appearing as a smaller positive wave after differentiation is complete, but this does not interfere with the identification of the W3 wave. When the stimulus intensity is further increased, the latency of each wave remains unchanged, and the amplitude increases. When the stimulus intensity exceeds 45 mA, the TSEP waveform no longer shows significant changes and becomes stable. For the V1 branch, regardless of the stimulus intensity, its W1 wave is significantly higher than that of other branches, and the differentiation of W2 and W3 waves is noticeably poorer than that of other branches. Therefore, this study considers that stimulus intensity <1 mA represents irregular subthreshold waveforms that are not TSEP, while 1 mA is the threshold stimulus for nerve excitation. The appropriate stimulus range for TSEP is 20–45 mA. The latencies of W1, W2, and W3 in TSEP waveforms elicited by stimulation of the main trunk, V1, V2, and V3 branches showed no statistically significant differences before and after MVD surgery (*p* > 0.05) (Figure 3).

### Amplitude and latency of each branch of TSEP

**Figure 4:**
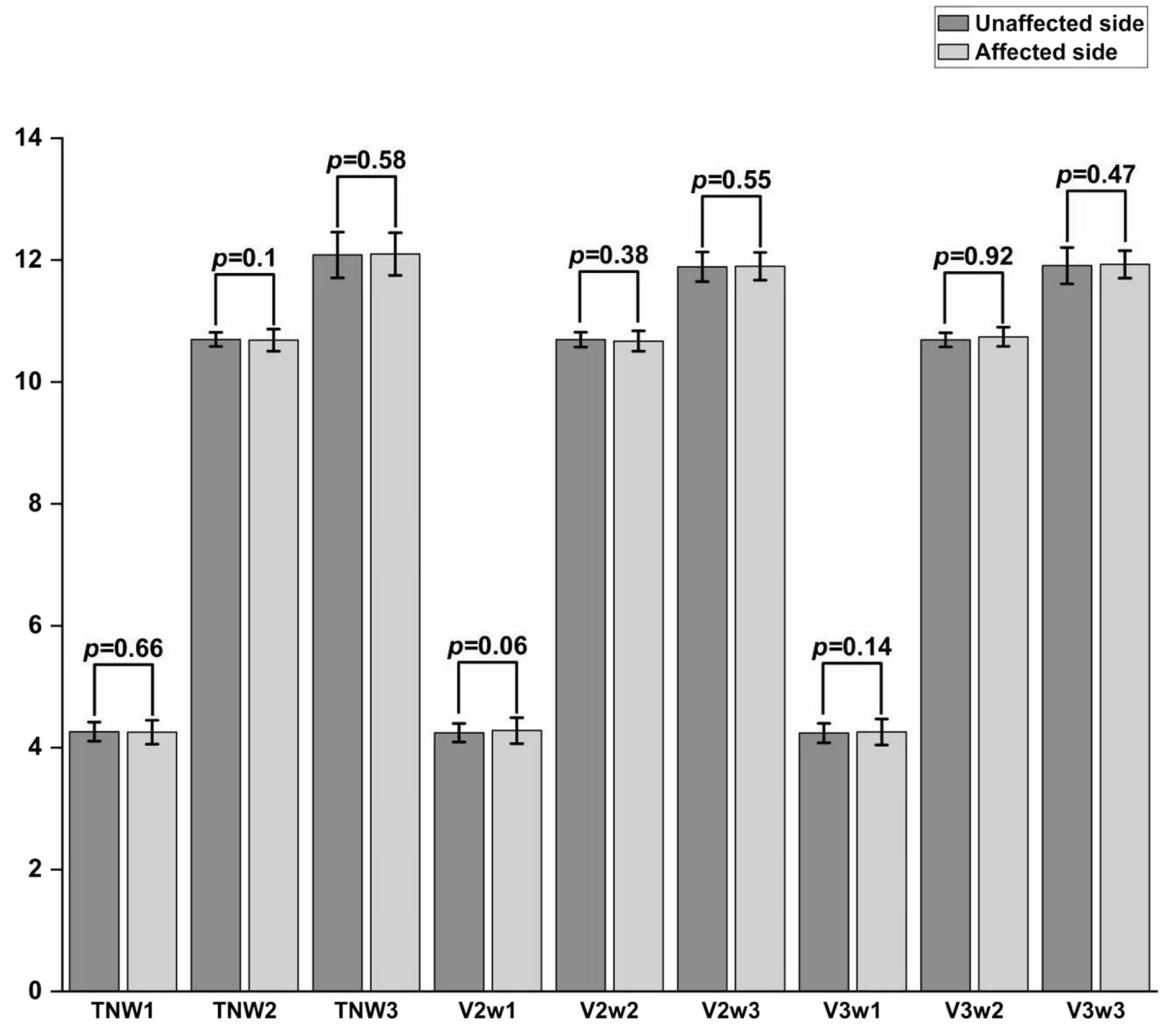
Comparison of TSEP amplitude between the unaffected and affected sides in patients with primary trigeminal neuralgia. *p < 0.05, **p < 0.01, ***p < 0.001; TN represents the trigeminal nerve; V1/V2/V3 represent the ophthalmic, maxillary, and mandibular branches of the trigeminal nerve, respectively.

**Figure 5:**
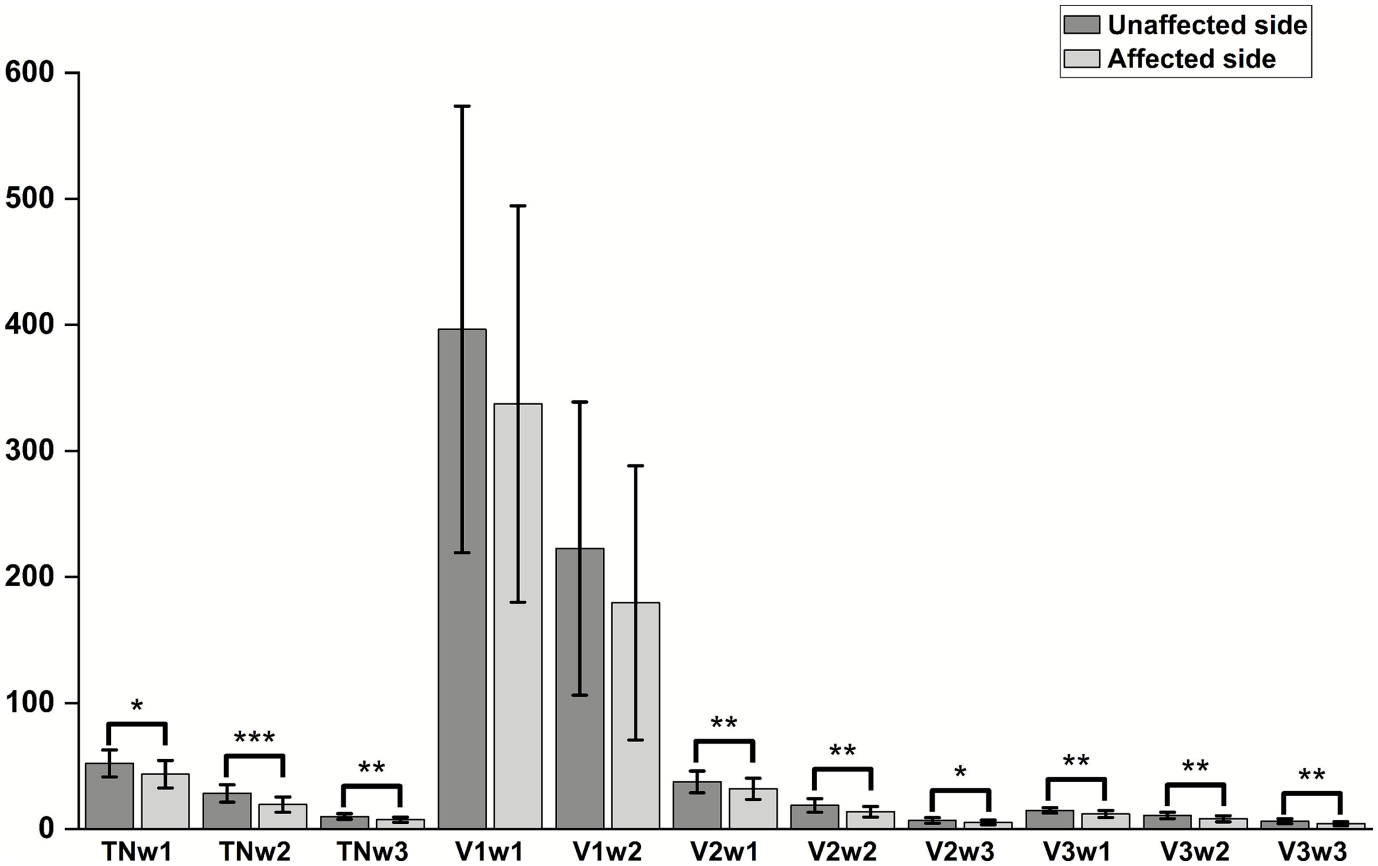
Comparison of TSEP latency before and after MVD surgery in patients with primary trigeminal neuralgia.

There were statistically significant differences in the W1, W2, and W3 amplitudes of the total TSEP, V2 branch, and V3 branch between the healthy side and the affected side in the study group (*p* < 0.05); however, there were no statistically significant differences in the W1 and W2 amplitudes of the V1 branch between the healthy and affected sides (*p* > 0.05), and in actual measurements, the W3 wave amplitude of V1 showed poor differentiation (Figure 4). There were no statistically significant differences in the latencies of W1, W2, and W3 of each branch in TSEP monitoring before and after MVD surgery (*p* > 0.05) (Figure 5).

### TSEP amplitude change rate

#### Analysis of the correlation between the amplitude change rate of each TSEP branch and postoperative pain level

**Figure 6:**
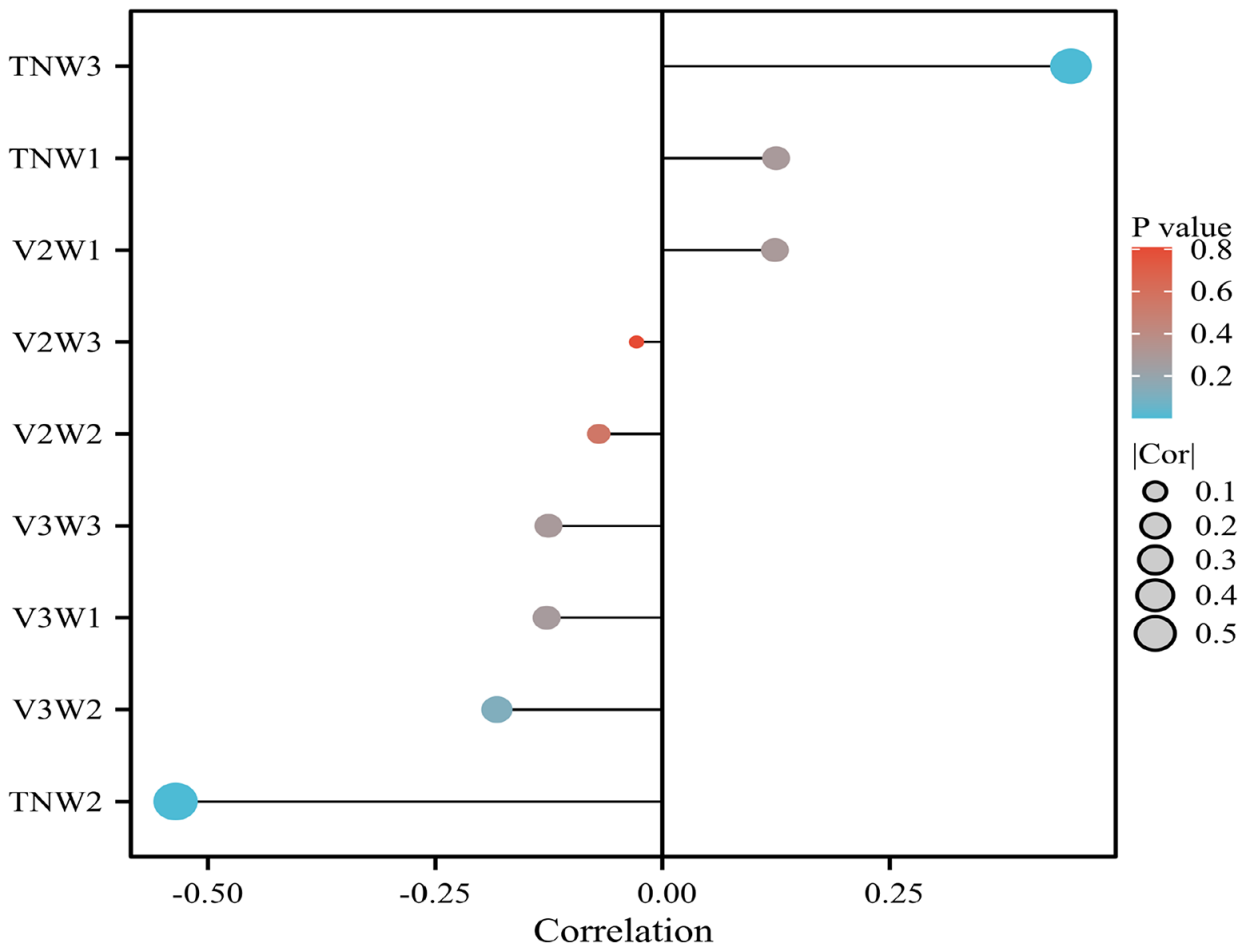
Analysis of the correlation between the rate of change in TSEP waveforms and postoperative prognosis.

Spearman correlation analysis was used to examine the relationship between the rate of change in amplitudes of various TSEP components and postoperative pain. It was found that the rate of change in the TNW2 amplitude of TSEP was strongly negatively correlated with postoperative pain (*r* = −0.563, *p* = 0.001), while the rate of change in the TNW3 amplitude was weakly positively correlated with postoperative pain (*r* = −0.460, *p* = 0.001) (Figure 6).

#### TNW2 and TNW3 predict surgical prognosis

**Figure 7:**
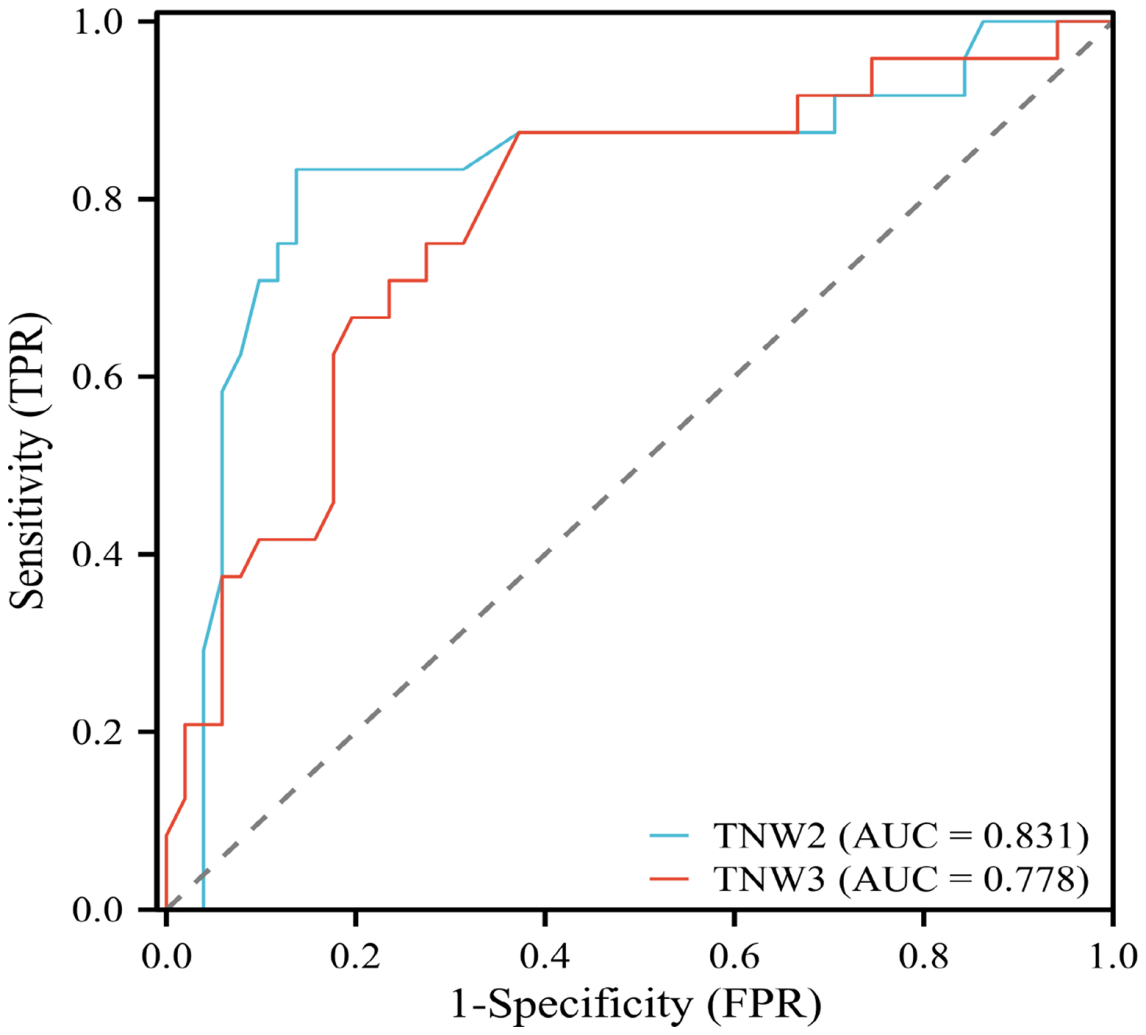
ROC curve of TNW2, TNW3 waveform change rate and MVD surgical prognostic efficacy evaluation.

In order to further study and analyze the significance of TSEP amplitude change rates in predicting postoperative prognosis after MVD, as well as to analyze the range of waveform change rates indicative of better prognosis, we used ROC (Receiver Operating Characteristic) curves to examine the relationship between TNW2, TNW3, and postoperative prognosis (Figure 7). TNW2 can indicate postoperative prognosis (AUC = 0.792, *p* = 0.001); the optimal cutoff value is 1.595. TNW3 can also indicate postoperative prognosis (AUC = 0.760, *p* = 0.001); the optimal cutoff value is 1.535 (Table 2).

**Table 2:** ROC curve analysis values of TNW2 and TNW3 waveform change rates and MVD surgical prognostic efficacy evaluation.

Amplitude growth percentage (%)	AUC	Cut-off value	Sensitivity	Specificity	*p*	95%CI	
						Lower limit	Upper limit
TNW2	0.831	1.595	0.833	0.863	0.001^**^	0.716	0.947
TNW3	0.718	1.535	0.875	0.727	0.001^**^	0.661	0.895

*p < 0.05, **p < 0.01, ***p < 0.001.

#### Cox regression analysis of independent risk factors and model prediction for postoperative prognosis of MVD

**Figure 8:**
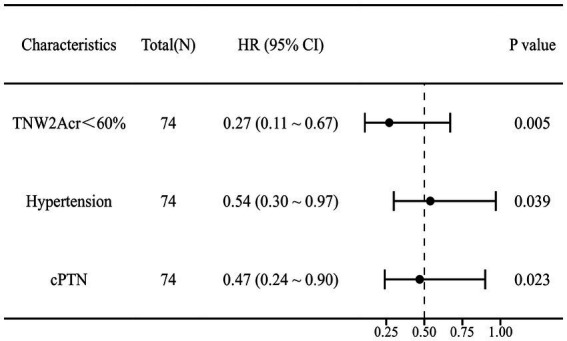
Multivariate Cox forest plot of independent factors contributing to poor postoperative prognosis of MVD in PTN patients. TNW2Acr represents the amplitude change rate of TNW2; CPTN represents the course of PTN disease.

**Figure 9:**
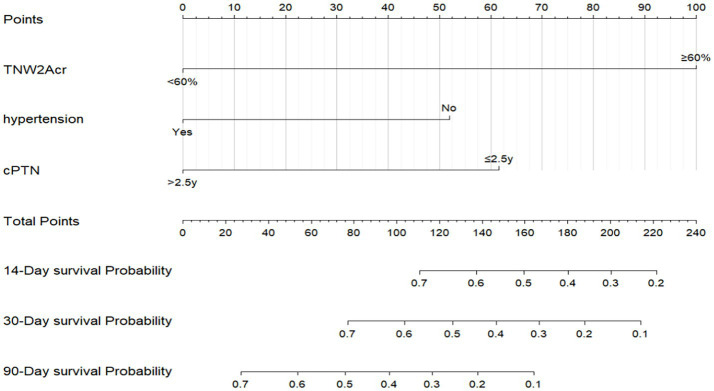
Prediction model for poor prognosis after MVD surgery in PTN patients. TNW2Acr represents the amplitude change rate of TNW2; CPTN represents the course of PTN disease.

**Figure 10:**
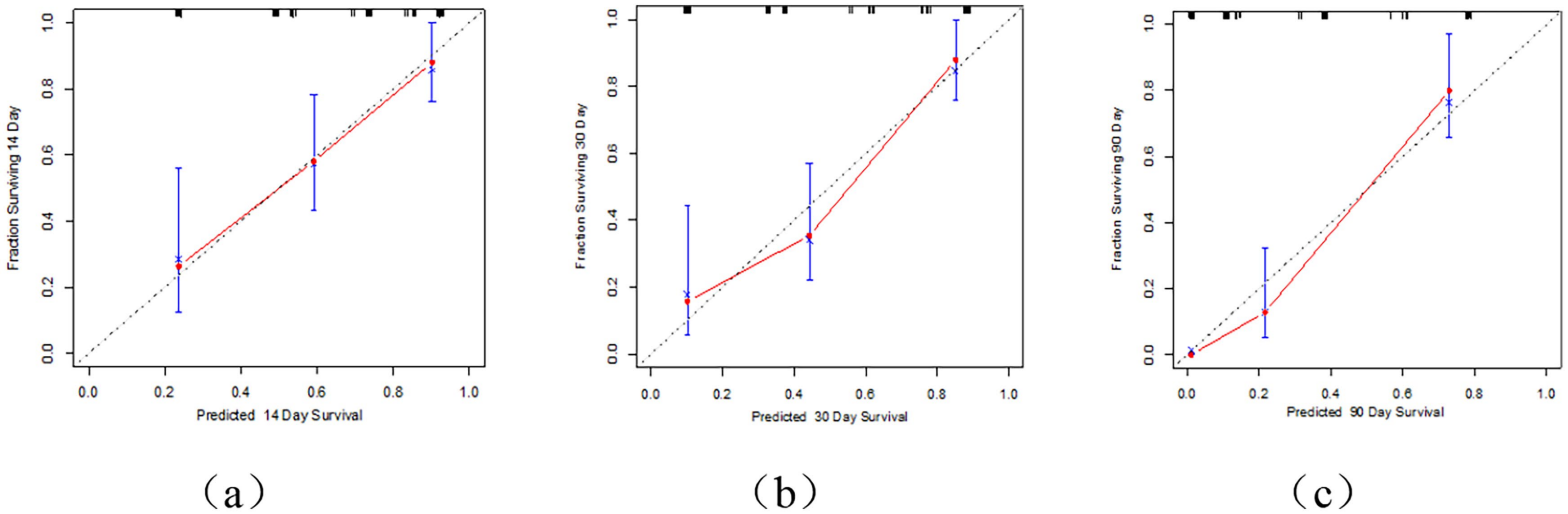
Calibration curve of MVD postoperative prognosis prediction model for PTN patients. **(a)** Represents the 14 day calibration curve; **(b)** represents the 30 day calibration curve; **(c)** represents the 90 day calibration curve.

This study employed univariate Cox regression analysis to investigate the association between various risk factors affecting the postoperative prognosis of patients with PTN undergoing MVD and the risk of poor postoperative prognosis. The results showed that a TNW2 amplitude change rate <60% (HR = 0.16, 95% CI: 0.07–0.35, *p* < 0.001) and a TNW3 amplitude change rate <50% (HR = 0.44, 95% CI: 0.22–0.85, *p* = 0.015) were significantly associated with the risk of the target event, indicating that a lower amplitude change rate may significantly increase the risk of the event. Additionally, the presence of comorbid hypertension (OR = 0.36, 95% CI: 0.20–0.63, *p* < 0.001), age (HR = 0.96, 95% CI: 0.94–0.98, *p* < 0.001), PTN duration (HR = 0.23, 95% CI: 0.13–0.42, *p* < 0.001), and preoperative pain score (HR = 0.90, 95% CI: 0.74–1.09, *p* = 0.279) were all significantly positively correlated with the risk of poor postoperative prognosis. However, gender (HR = 1.06, 95% CI: 0.58–1.95, *p* = 0.840) and diabetes (HR = 1.54, 95% CI: 0.66–3.64, *p* = 0.320) did not show statistical significance, suggesting that their association with the risk of the target event may be weak or nonexistent. The above results indicate that TNW2 and TNW3 amplitude change rates, hypertension, age, and PTN duration are important predictors of poor postoperative prognosis in patients with PTN undergoing MVD (Table 3). Incorporating factors with *p* < 0.1 from the univariate COX regression results into the multivariate COX regression analysis, it was indicated that TNW2 amplitude change rate < 60% (HR = 0.27, 95% CI: 0.11–0.67, *p* = 0.005), hypertension (HR = 0.54, 95% CI: 0.30–0.97, *p* = 0.039), and PTN duration (HR = 0.47, 95% CI: 0.24–0.90, *p* = 0.023) were independent prognostic factors associated with poor postoperative prognosis in patients with PTN undergoing MVD (Figure 8). Based on this, a predictive model was established. The calibration curve results indicated that the model exhibited good predictive ability at 14 days, 30 days, and 90 days (Figures 9, 10).

**Table 3:** Univariate Cox analysis of risk factors for poor postoperative prognosis of MVD in PTN patients.

Variable	β	S.E	*Z*	*P*	HR (95%CI)
Age	−0.04	0.01	−3.37	<0.001^***^	0.96 (0.94–0.98)
Gender	0.06	0.31	0.20	0.840	1.06 (0.58–1.95)
TNW2 amplitude change rate < 60%	−1.86	0.41	−4.50	<0.001^***^	0.16 (0.07–0.35)
TNW3 amplitude change rate<50%	−0.83	0.34	−2.43	0.015^*^	0.44 (0.22–0.85)
Hypertension	−1.04	0.29	−3.58	<0.001^***^	0.36 (0.20–0.63)
PTN course	−1.47	0.31	−4.78	<0.001^***^	0.23 (0.13–0.42)
Preoperative pain score	−0.11	0.10	−1.08	0.279	0.90 (0.74–1.09)
Diabetes	0.43	0.44	1.00	0.320	1.54 (0.66–3.64)

*p < 0.05, **p < 0.01, ***p < 0.001.

**Figure 11:**
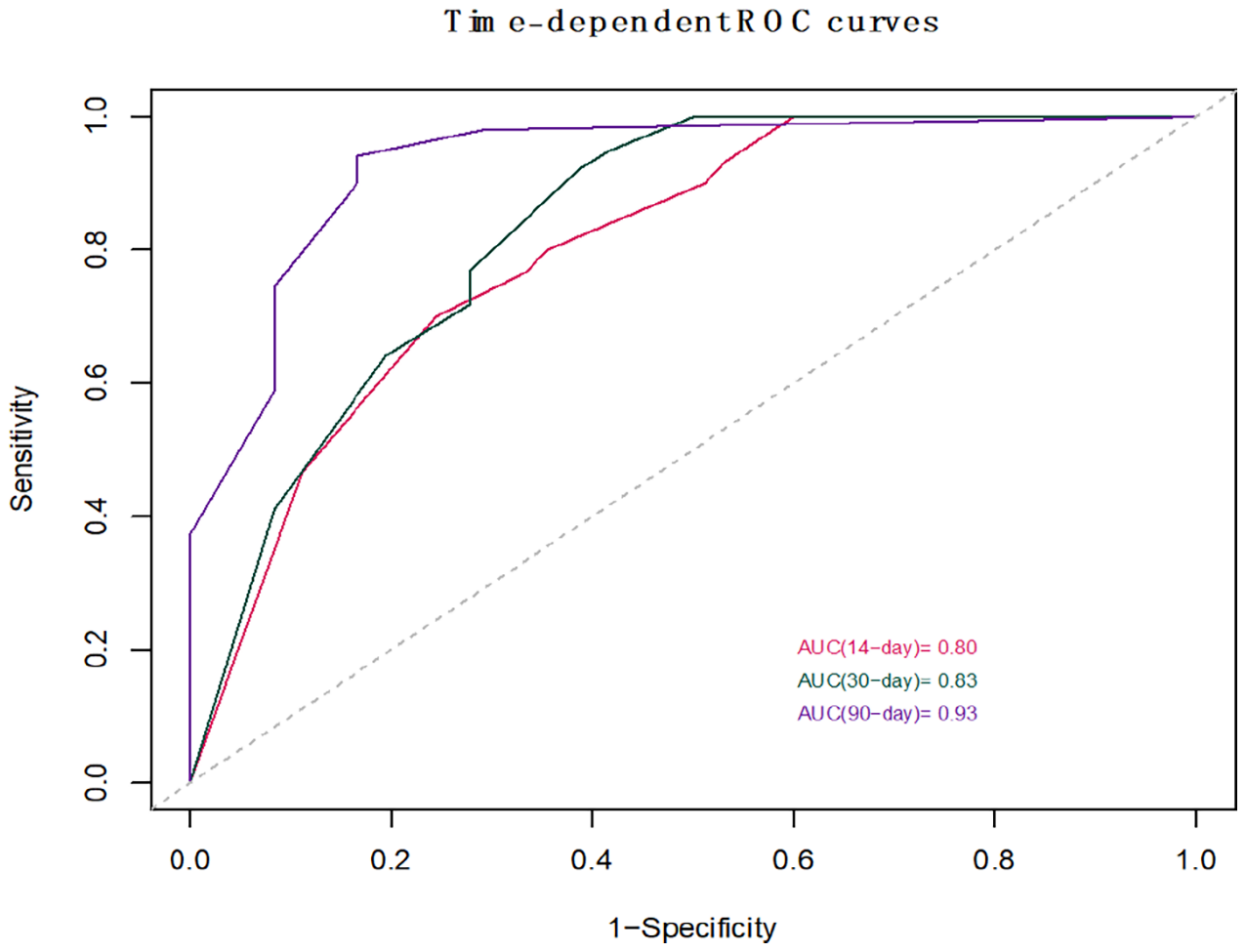
Calibration curve of MVD postoperative prognosis prediction model for PTN patients.

**Figure 12:**
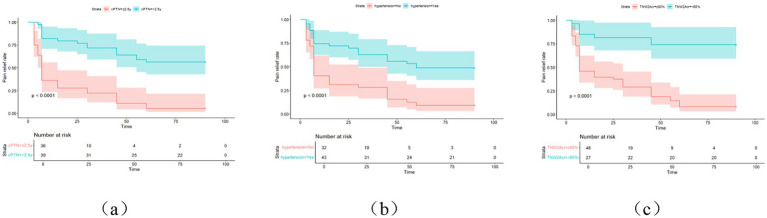
KM curve of painless survival after MVD surgery in PTN patients. cPTN represents the course of PTN, and **(a)** represents the postoperative survival curve of PTN course; **(b)** represents the postoperative generation curve of combined hypertension; **(c)** represents the TNW2 amplitude change rate generation curve line.

The Receiver Operating Characteristic (ROC) curve analysis revealed AUC values of 0.80, 0.83, and 0.93 at 14 days, 30 days, and 90 days, respectively, indicating good discriminatory power of the model. As time progressed, the AUC gradually improved, suggesting that with longer observation periods, it becomes easier to distinguish clinical differences in pain outcomes (Figures 4, 5). Kaplan–Meier survival analysis further confirmed that TNW2, hypertension, and PTN were significantly associated with prognosis (Log-rank test, all *p* < 0.001) (Figures 11, 12).

## Discussion

Trigeminal somatosensory evoked potential (TSEP), as an electrophysiological monitoring technique, reflects cortical electrical activity and can indicate the function of the trigeminal nerve, trigeminal nuclei, and trigeminal pathways. It effectively responds to excitability changes in the peripheral nerve-cortical circuit (15). Leandri et al. believe that TSEP may correspond to the functional recovery of the trigeminal nerve after microvascular decompression (MVD) surgery (16). Chappa et al.’s research shows that the stimulation intensity required to elicit a short-latency somatosensory evoked potential can only excite the thicker myelinated fibers in peripheral nerves. To excite thinner nerve fibers, a much higher stimulation intensity is needed. The fibers that transmit pain sensation are type III thin myelinated fibers and type IV unmyelinated fibers. Even with needle electrodes, it is difficult to excite these fibers without direct nerve stimulation (17, 18). Therefore, the key to stably inducing TSEP lies in the selection of the puncture site for the stimulating electrode. The puncture needle should penetrate the supraorbital foramen (notch), infraorbital foramen, and mental foramen to directly stimulate the supraorbital nerve, infraorbital nerve, and mental nerve, respectively, in order to induce a waveform. This requires high puncture skills from electrophysiologists, who must ensure precise puncture while avoiding nerve damage. Although most previous studies have conducted detailed research on the waveform of TSEP using this puncture technique, which has contributed to a deeper understanding of the principles of TSEP, this has increased the complexity of clinical use and is inconsistent with the goal of simplicity and timeliness during surgery. This study, by setting the operation mode and monitoring parameters of TSEP, has achieved for the first time no significant change in the latency of TSEP before and after surgery, with no significant statistical difference. Instead, it relies solely on the rate of amplitude change in TSEP to assess the decompression situation during MVD surgery and the short-term prognosis in patients with trigeminal neuralgia (PTN).

This study found that among the waveforms of the general branch, ophthalmic branch, maxillary branch, and mandibular branch of the trigeminal nerve monitored by TSEP, the changes in the general branch of TSEP were the most pronounced. Regardless of the painful site in any of these branches, significant waveform changes were ultimately detected in the general branch. This may be related to the conduction of TSEP stimulation potentials. When peripheral nerves are stimulated, evoked potentials are transmitted through the trigeminal ganglion to the trigeminal nucleus in the brainstem. Then, the information is sent to the ventroposterior medial thalamic nucleus, stimulating the posterior central gyrus of the parietal lobe (7, 8). That is, regardless of which branch is stimulated, the stimulation potential must pass through the general branch of the trigeminal nerve. Furthermore, through correlation analysis and COX regression analysis, this study found that the amplitude changes in the general branch of TSEP were the most pronounced, with the strongest correlation between the rate of amplitude change in TNW2 and postoperative pain scores, followed by TNW3; and a rate of amplitude change in TNW2 < 60% was an independent risk factor for poor prognosis in patients with PTN after MVD surgery. These may be related to the pathophysiological mechanisms underlying the onset of PTN. Trigeminal neuralgia is primarily caused by compression of the trigeminal nerve root by blood vessels. Since the trigeminal nerve root has a demyelinating zone at the entry zone into the brainstem (REZ), which is a transitional zone between the central and peripheral nervous systems and is sensitive to vascular compression, long-term vascular compression can lead to demyelinating lesions in this area, reducing the excitability threshold of nerve fibers. This results in abnormal conduction between non-pain fibers (such as Aβ fibers) and pain fibers (such as Aδ fibers), causing the efferent impulses from the central nervous system to be converted into afferent impulses, leading to the outbreak of pain (19, 20). Previous studies on the origin of each wave in TSEP have indicated that W1 originates from the semilunar ganglion of the trigeminal nerve; W2 originates from the REZ; W3 originates from the main sensory nucleus of the trigeminal nerve; N5 originates from the trigeminal-thalamic tract, and P6 originates from the thalamus. The key research objects are W2, which reflects the electrical conduction of the trigeminal REZ, and W3, which reflects the electrical changes in the main sensory nucleus of the trigeminal nerve (13, 14). Studies have also shown that the latency and amplitude of W2 and W3 in TSEP can serve as reliable and safe references for monitoring the impact of thermal metabolism during radiofrequency thermocoagulation of ganglia in patients with idiopathic trigeminal neuralgia. Leandri et al. also used changes in W2 in TSEP to monitor the extent of lesions (16, 21). These conclusions all demonstrate the rationality of using TNW2 and TNW3 as our primary observation objects.

Therefore, in further research, we found that TNW2 and TNW3 can not only serve as diagnostic criteria for assisting in the diagnosis of PTN, but their waveform change rates also indicate the prognosis of PTN patients after MVD surgery. Through correlation analysis, we discovered a correlation between the amplitude change rates of TNW2 and TNW3 and the degree of postoperative pain, that is, the greater the amplitude change rate, the less severe the postoperative pain. Further analysis of the relationship between amplitude change rate and postoperative pain through receiver operating characteristic (ROC) curves revealed that the optimal cutoff value for the amplitude change rate of TNW2 is 1.595 (i.e., 59.5%, approximately 60%), and the optimal cutoff value for the amplitude change rate of TNW3 is 1.535 (i.e., 53.5%, approximately 50%). In summary, we set the criteria that TNW2 < 60% and TNW3 < 50% for the amplitude change rates of W2 and W3 waves in TSEP before and after MVD surgery in PTN patients indicate a poor short-term prognosis after surgery. This conclusion was incorporated into univariate Cox regression analysis, and with a *p*-value < 0.1 as the screening criterion, it was further analyzed in multivariate Cox regression, where TNW2 < 60% was found to be an independent factor affecting the poor short-term prognosis of PTN patients after MVD surgery.

This study employed the Trigeminal Somatosensory Evoked Potential (TSEP) monitoring technique to systematically investigate the correlation between intraoperative TSEP amplitude change rate and the prognosis of patients with trigeminal neuralgia (PTN) after microvascular decompression (MVD). Through Cox regression analysis, a prognostic prediction model for PTN patients undergoing MVD surgery was constructed, integrating electrophysiological parameters (TNW2 amplitude change rate) and clinical variables (hypertension, PTN duration). Multivariate analysis revealed that a TNW2 amplitude change rate <60% (HR = 0.27, 95% CI: 0.11–0.67), hypertension (HR = 0.54, 95% CI: 0.30–0.97), and PTN duration ≥2.5 years (HR = 0.47, 95% CI: 0.24–0.90) were independent risk factors for poor prognosis after MVD. Both hypertension and PTN duration may delay postoperative functional recovery. This is consistent with previous reports: long-term hypertension may lead to decreased vascular elasticity, exacerbating nerve compression. Prolonged PTN duration is associated with demyelinating lesions in the trigeminal nerve entry zone (REZ). Numerous neuroelectrophysiological, neuroimaging, and histological studies have confirmed that demyelinating lesions of the trigeminal nerve caused by NVC are the primary pathogenic factor. MVD can relieve compression, interrupt the PTN pain-generating mechanism, and even reverse demyelinating lesions, restoring normal physiological conditions. However, the local demyelinating lesions of the trigeminal nerve caused by NVC are a slow process, often requiring years or longer to cause pathophysiological changes; this also explains why some individuals with NVC do not exhibit clinical symptoms of facial pain (22, 23). The degree of demyelinating lesions and the duration of the lesions in the trigeminal nerve after decompression through MVD also affect the length of time required to reverse its hypersensitive or diseased state, indicating that there are differences in the degree and duration of pain relief among different patients undergoing MVD surgery.

In addition, a prognostic prediction model combining electrophysiological and clinical features was constructed based on Cox regression. This model predicts the probability of changes in facial pain severity over time in PTN patients after undergoing MVD surgery, exhibiting high discrimination at different postoperative time points (14 days, 30 days, and 90 days) (AUC values of 0.80, 0.83, and 0.93, respectively). Although the follow-up period for PTN patients included in this study was relatively short (1–90 days), studies have shown that 12.5% of PTN patients experienced a decline in efficacy or pain recurrence at 6 months after undergoing trigeminal nerve MVD surgery. Furthermore, incomplete pain relief in PTN patients shortly after MVD surgery may be associated with poor long-term prognosis. Regarding long-term prognosis, studies have found that as many as 16.1–21.4% of PTN patients fail to achieve sustained satisfactory efficacy 1–2 years after MVD surgery (24). Therefore, this model models the early postoperative pain changes in PTN patients after MVD surgery, predicting the probability of early postoperative pain changes in PTN patients. Our research data has the advantage of predicting early postoperative prognosis in PTN patients. The nomogram model proposed in this study integrates TSEP features (TNW2 amplitude change rate) and clinical variables (hypertension, PTN duration), enabling individualized assessment of patients’ postoperative pain relief probability. This tool is particularly suitable for intraoperative real-time decision-making: if the TNW2 amplitude change rate is less than 60%, the surgeon can adjust the decompression range to improve prognosis. Additionally, the high discrimination ability of the model (90-day AUC = 0.93) indicates its potential value in surgical risk stratification, helping to screen high-risk patients who require close follow-up.

### Clinical significance and innovation

This study groundbreakingly proposes an intraoperative monitoring strategy centered on the TNW2 amplitude change rate: when the TNW2 amplitude change rate is less than 60%, the surgeon can immediately adjust the decompression range to optimize the therapeutic effect. Furthermore, the nomogram model achieves individualized prediction of postoperative pain relief probability by integrating easily accessible clinical and electrophysiological parameters. Compared to previous studies that only focused on overall relief rates or recurrence rates, the dynamic predictive ability of this model (increasing AUC gradient over time) better meets clinical decision-making needs and provides an important tool for precision medicine in TN.

### Limitations and future directions

This study has the following limitations: Firstly, the sample size is small (*n* = 64) and the follow-up period is short (3 months), which fails to assess the long-term recurrence risk; secondly, the model does not incorporate imaging or molecular markers, which may affect the comprehensiveness of prediction; finally, the single-center design limits the generalizability of the conclusions. In the future, it is necessary to verify the external validity of the model through multi-center, large-sample cohort studies, and explore the combined application of TSEP with other biomarkers (such as neuroinflammatory factors). In addition, extending the follow-up period to over 1 year will help clarify the predictive value of TSEP for long-term efficacy.

## Conclusion

This study simplifies the TSEP monitoring method (requiring only puncture of muscle groups) and reduces the difficulty of interpretation (effectively predicting prognosis solely through amplitude change rate), significantly enhancing the clinical practicality of TSEP. Intraoperative TSEP monitoring (especially the amplitude change rate of TNW2) can guide MVD procedures in real-time and predict short-term postoperative prognosis. The nomogram model constructed based on the duration of hypertension and PTN exhibits high accuracy, providing a new strategy for individualized treatment of TN patients. Future research needs to further optimize monitoring techniques and expand the dimensionality of the model to promote the development of precision medicine for TN.

## Data Availability

The raw data supporting the conclusions of this article will be made available by the authors, without undue reservation.
